# Geographic clustering of elevated blood heavy metal levels in pregnant women

**DOI:** 10.1186/s12889-015-2379-9

**Published:** 2015-10-09

**Authors:** Katherine E. King, Thomas H. Darrah, Eric Money, Ross Meentemeyer, Rachel L. Maguire, Monica D. Nye, Lloyd Michener, Amy P. Murtha, Randy Jirtle, Susan K. Murphy, Michelle A. Mendez, Wayne Robarge, Avner Vengosh, Cathrine Hoyo

**Affiliations:** Biodemography of Aging Research Unit (BARU), Duke University, Room A110C, Erwin Mill Building, 2024 W. Main St, Durham, NC 27708 USA; Division of Water, Climate, and the Environment, School of Earth Sciences, The Ohio State University, 275 Mendenhall Laboratory, 125th South Oval, Columbus, OH USA; Center for Geospatial Analytics, North Carolina State University, 5125 Jordan Hall, Campus Box 7106, Raleigh, NC USA; Department of Biological Sciences, and Center for Human Health and the Environment, North Carolina State University, 850 Man Campus Dr, Campus Box 7633, Raleigh, NC USA; Biological Sciences, University of North Carolina at Charlotte, 9201 University City Blvd, Charlotte, NC USA; Department of Community and Family Medicine, Duke University, 2200 Main St, Durham, NC USA; Department of Obstetrics and Gynecology, Duke University, 2608 Erwin Rd, Suite 210, Durham, NC USA; Department of Nutrition, University of North Carolina, 101 Manning Dr, Chapel Hill, NC USA; Department of Soil Science, North Carolina State University, PO Box 7619, Raleigh, NC USA; Nicholas School of the Environment, Duke University, 450 Research Dr, Durham, NC USA

**Keywords:** Cadmium, Lead, Mercury, Arsenic, Spatial analysis

## Abstract

**Background:**

Cadmium (Cd), lead (Pb), mercury (Hg), and arsenic (As) exposure is ubiquitous and has been associated with higher risk of growth restriction and cardiometabolic and neurodevelopmental disorders. However, cost-efficient strategies to identify at-risk populations and potential sources of exposure to inform mitigation efforts are limited. The objective of this study was to describe the spatial distribution and identify factors associated with Cd, Pb, Hg, and As concentrations in peripheral blood of pregnant women.

**Methods:**

Heavy metals were measured in whole peripheral blood of 310 pregnant women obtained at gestational age ~12 weeks. Prenatal residential addresses were geocoded and geospatial analysis (Getis-Ord G_i_* statistics) was used to determine if elevated blood concentrations were geographically clustered. Logistic regression models were used to identify factors associated with elevated blood metal levels and cluster membership.

**Results:**

Geospatial clusters for Cd and Pb were identified with high confidence (*p*-value for G_i_* statistic <0.01). The Cd and Pb clusters comprised 10.5 and 9.2 % of Durham County residents, respectively. Medians and interquartile ranges of blood concentrations (μg/dL) for all participants were Cd 0.02 (0.01–0.04), Hg 0.03 (0.01–0.07), Pb 0.34 (0.16–0.83), and As 0.04 (0.04–0.05). In the Cd cluster, medians and interquartile ranges of blood concentrations (μg/dL) were Cd 0.06 (0.02–0.16), Hg 0.02 (0.00–0.05), Pb 0.54 (0.23–1.23), and As 0.05 (0.04–0.05). In the Pb cluster, medians and interquartile ranges of blood concentrations (μg/dL) were Cd 0.03 (0.02–0.15), Hg 0.01 (0.01–0.05), Pb 0.39 (0.24–0.74), and As 0.04 (0.04–0.05). Co-exposure with Pb and Cd was also clustered, the *p*-values for the G_i_* statistic for Pb and Cd was <0.01. Cluster membership was associated with lower education levels and higher pre-pregnancy BMI.

**Conclusions:**

Our data support that elevated blood concentrations of Cd and Pb are spatially clustered in this urban environment compared to the surrounding areas. Spatial analysis of metals concentrations in peripheral blood or urine obtained routinely during prenatal care can be useful in surveillance of heavy metal exposure.

## Background

The metals cadmium (Cd), mercury (Hg), arsenic (As) and lead (Pb) are ubiquitous environmental pollutants that persist in the environment for long periods. Reducing chronic human exposure to these contaminants and their effects is a high priority for the Environmental Protection Agency (EPA) and the Agency for Toxic Substances and Disease Registry (ATSDR). Common sources of chronic exposure to Cd and Pb for non-smokers are dietary, via ingestion of contaminated staples (lettuce, spinach, potatoes, and grains), contaminated water via old housing pipes for Pb, or seafood for As and Hg [1, 2]. Because these metals are ubiquitous in the soils of many urban landscapes [3–5], they are transported indoors, become part of house dust and are either ingested or inhaled.

In cross-sectional studies, whether measured in urine or peripheral blood, chronic exposure to one or more heavy metals elicits a broad range of dysfunctions targeting many organ systems [6, 7]. In humans, these metals have been implicated in common chronic diseases including cerebrovascular and cardiovascular disease [8], diabetes [9], skeletal disorders [10, 11], renal disorders [12, 13], and cancer [14–18]. Some studies suggest heavy metal effects may be sex or race specific [19]. Fetal exposure to Hg, Cd, Pb and As is associated with lower birth weight [20–23], shorter birth length [21], and childhood obesity [23–31]. Low birth weight, whether due to preterm birth or growth restriction, is a consistent risk factor for rapid early postnatal growth, childhood obesity or cardiometabolic disorders, even in the absence of obesity [32, 33]. Both low birth weight and many of these conditions and diseases disproportionately affect ethnic minority populations. Blood concentrations at which health effects may increase substantially are still unknown, and are an active topic of investigation; however reportable values range from 0.5–1 μg/dL for Cd, 0.5–1.5 μg/dL for Hg, 5–30 μg/dL for Pb, and 7 μg/dL for As [34–41].

Existing risk assessment strategies are un-scalable as population-wide public health measures as they are triggered by a known point source or clustered diseases or conditions, yet low dose exposure may elicit no immediate symptoms. Interventions that include dietary manipulation to either reduce absorption (e.g. iron and Cd) or increase excretion (e.g. As and one carbon cycle nutrients) [42–44] have been proposed. However, safety margins are narrow and human data are limited. In this study, we use geospatial analysis to determine if elevated blood concentrations of heavy metals are geographically clustered using routinely collected peripheral blood concentrations of pregnant women. We also use logistic regression to identify factors associated with elevated blood metal levels and cluster membership and present soil and water measurements of Cd and Pb.

## Methods

### Study participants

Between 2009 and 2011, 1700 participants were recruited from six prenatal clinics that use Duke Obstetrics and Durham Regional Hospital Obstetrics, as part of the Newborn Epigenetic STudy (NEST). Details of participant accrual have been previously described [45, 46]. Response rate was 66.7 %. Eligible women were aged 18+ years, pregnant and using one of 6 prenatal clinics in Durham County and intending to use one of two participating obstetric facilities in Durham County for delivery—the catchment area for Duke affiliated prenatal clinics is Durham and contiguous counties. From these, we excluded women with an established HIV infection and women who planned to relinquish custody of the index child or move states before delivery. Four metals were measured in 310 women living in Durham and contiguous counties. The geo-spatial analyses described here are limited to the 239 mothers who lived within Durham County during the prenatal period and had geocodable addresses. For Pb analyses we also limited the analyses to women with Pb values <10 μg/dL. Women included in these analyses are comparable to the cohort from which the sample derived with respect to maternal race, cigarette smoking during pregnancy, BMI before pregnancy, parity, delivery route, and education (*p >* 0.05). The study protocol for data and specimen collection and analysis was approved by the University Institutional Review Boards (IRB) at Duke and North Carolina State Universities. Participants gave written informed consent.

### Data and specimen collection

At study enrollment, maternal peripheral blood samples were collected at a mean gestational age of 12 weeks. Self- or interviewer-administered questionnaires were used to collect covariate data that included maternal race, education, cigarette smoking, parity, and obesity status before pregnancy. Trained phlebotomists obtained 10 ml of peripheral blood using EDTA tubes, 1 ml of which was stored whole. Medical records were used to obtain parturition data.

#### Measurement of metals in human blood

Frozen whole peripheral blood samples obtained from women were transferred to Duke School of the Environment, equilibrated to room-temperature, homogenized and ~0.2 mL aliquots were pipetted into a trace-metal-clean test tubes and verified gravimetrically to ±0.001 mg using a calibrated mass balance as previously described [47–50]. Samples were then spiked with internal standards consisting of known quantities (10 and 1 ng/g, respectively) of indium (In) and bismuth (Bi) (obtained from SCP Science), used to correct for instrumental drift. The solutions were then diluted using water purified to 18.2 MΩ/cm resistance (by a Milli-Q water purification system, Millipore, Bedford, Mass., USA) and acidified using ultra-pure 12.4 mol/L hydrochloric acid to result in a final concentration of 2 % hydrochloric acid (by volume). All standards, including aliquots of the certified NIST 955c, and procedural blanks were prepared by the same process.

Concentrations of metals Cd, Pb, Hg, As were measured in nanograms per gram of blood weight, and then converted to μg/dL blood volume, to facilitate comparison with other studies. We used a Perkin Elmer DRC II (Dynamic Reaction Cell) axial field inductively coupled plasma mass spectrometry (ICP-MS) at the University of Massachusetts-Boston [47–50]. To clean and reduce memory effects, sample lines were sequentially washed with 18.2 MΩ cm resistance (by a Milli-Q water purification system, Millipore, Bedford, Mass., USA) water for 90 s and a 2 % nitric acid solution for 120 s between analyses. Procedural blanks were analyzed within each block of 10 samples, to monitor and correct for instrumental and procedural backgrounds. Calibration standards used to determine metals in blood included aliquots of 18.2 MΩ cm resistance H_2_O, NIST 955c SRM, and NIST 955c SRM spiked with known quantities of each metal in a linear range from 0.025 to 10 ng/g. Standards were prepared from 1000 mg/L single element standards obtained from SCP Science, USA. Method detection limits were calculated according to the two-step approach using the t_99_S_LLMV_ method (USEPA, 1993) at 99 % CI (t = 3.71). The MDLs yielded values of 690, 58, 157, 48 pg/g for As, Cd, Hg, and Pb, respectively. Limits of quantification (LOQ) and limits of detection (LOD) and according to Long and Winefordner (1983) were less than 71 pg/g and 210 for As, 6.9 pg/g and 23 pg/g for Cd, 16 pg/g and 49 pg/g for Hg, and 5.9 pg/g and 17.2 pg/g for Pb.

#### Measurement of Pb and Cd in soil and water samples

To identify potential sources of exposure, top soil and water samples collected from 24 households of women with Cd levels ≥ 0.1 ng/g (0.01035 μg/dL) and 17 samples with Cd levels < 0.1 ng/g. We employed standard procedures where 3–5 samples were taken from a 1 square foot area from the street-side of participants’ homes. Soil testing used a modified soil index extraction with 1.0 M Optima grade hydrochloric acid by the NC State soil science laboratory to account for the solubility of Pb and Cd in soils, known to be pH dependent. Extraction procedure involved 10 g of ground (ceramic mortar and pestle) and sieved sample (1 mm stainless steel sieve) to which was added 30 mL of 1.0 M HCl in a 50 mL PVC disposable centrifuge tube with HDPE screw cap lid. The sealed centrifuge tube was inverted several times to fully wet the sample aliquot, and then sets of tubes were placed on a reciprocating shaker for a total of 1 h. The tubes were removed from the shaker and inverted several times to promote total contact of the sample aliquot with the extracting solution every 15 min, then allowed to stand for 10 min to settle larger particles, then centrifuged at 3000 rpms (International Model K centrifuge) for 30 min. The cleared supernatant was decanted and filtered through Whatman 42 filter paper to remove floating debris and remaining fine particulates disturbed during the decanting step. The filtered extract was captured in a clean PVC centrifuge tube with HDPE screw cap lid. Blanks were carried through the extraction procedure and the final filtration step.

Analysis of the filtered extractions was carried out using a Perkin Elmer Model 2000 DV ICP-Emission Spectrometer equipped with a Meinhart nebulizer. Three separate analytical lines were monitored to account for the high background matrix of iron, aluminum and calcium mobilized by the use of the 1.0 M HCl extract. Where necessary, multispectral background correction was used to remove interferences and to achieve agreement between all three analytical lines. Calibration of the ICP emission spectrometer was done with NIST-traceable stock standards in a background matrix of 1.0 M HCl Optima-grade acid [51].

### Statistical analysis

#### Cluster mapping and analysis

Of the 239 women living within the county boundaries, we excluded three participants with extremely high circulating blood Pb concentrations, that may unduly influence our findings (69.64, 109.17, 259.39 μg/dL) and set three values below detectable limit to ‘0’. Cluster mapping and analysis was performed using the Hot Spot Analysis and Kernel Density Tools within ArcGIS 10.2.2 [52]. For ethical reasons, heat maps were generated from the point-level data for maternal blood metal concentrations to de-identify the specific location of study participants with their measured concentrations. Two participants with negative values deriving from data normalization were also removed from the analysis. The concentration density was calculated over a 1-km^2^ grid and reported as μg/dL/km^2^. To identify potential clusters, the Getis-Ord Gi* statistic was calculated for each data point and for each metal. This statistic is based on the attribute value for the metal concentration, a spatial weight between neighboring features, and the total number of features. The Gi* statistic generates a Z-score and corresponding *p-*value to test the hypothesis that a given pattern is the result of randomness in the data. For this analysis, a fixed-distance band of 1500 m was used to calculate the spatial influence of neighboring points. Points falling outside the 1500 m band did not influence the calculation of the Gi*. The resulting significance level (*p-*value) was then plotted using a heat map to show high (*p <* 0.01) and low cluster probabilities for a given metal.

#### Regression modeling

We used logistic regression models to identify risk factors for blood metal levels (categorized into tertiles) and cluster membership using ‘clusters’ or ‘hot-spots’ identified by Getis-Ord Gi* statistics. Blood metal levels were categorized into tertiles because the natural log did not normalize the distribution of data. Covariates considered as potential confounders were maternal years of education, an indicator of socioeconomic status; maternal pre-pregnancy obesity estimated as body mass index (BMI); maternal whole blood folate concentration, a commonly consumed and measured one-carbon cycle nutrient during pregnancy which also inversely associated with As levels and perhaps other metals; maternal smoking status, an alternate source of heavy metal exposure, categorized as smoked during pregnancy (yes/no); and physical activity (any/none), measured using the validated Pregnancy Physical Activity Questionnaire [53]. These factors have previously been shown to predict elevated metal levels [54].

## Results

Table 1 shows the distribution of sociodemographic and lifestyle characteristics of the 310 participants included in the analyses. Non-Hispanic African Americans comprised 35.5 % of the study population, and Hispanics, non-Hispanic Whites, and non-Hispanic Others comprised 31.6, 29.0 and 3.9 %, respectively. About one third were younger than 25 years; over half the sample had a high school education level or less. Approximately three-quarters reported any physical activity, and first trimester blood folate levels averaged 217.7 mcg/L (range 25.1 to 358.1). Fifteen percent reported smoking during pregnancy and 57.7 % were overweight, obese, or extremely obese (31.9, 14.7, or 11.1 % respectively). The majority of offspring (82 %) had a birth weight of 2500 to 4000 g and approximately 90 % were born at term.

**Table 1:** Characteristics of pregnant women and blood metals levels (μg/dL)

			Cadmium (Cd)		Mercury (Hg)		Lead (Pb)		Arsenic (As)	
	N	%	Geometric Mean^a^	Median (IQR)	Geometric Mean^a^	Median (IQR)	Geometric Mean^a^	Median (IQR)	Geometric Mean^a^	Median (IQR)
Overall	310	-	0.03	0.02 (0.01–0.04)	0.03	0.03 (0.01–0.07)	0.38	0.34 (0.16–0.83)	0.05	0.04 (0.04–0.05)
in Cd Cluster	25	8.1 %	0.05	0.06 (0.02–0.16)	0.02	0.02 (0.00–0.05)	0.51	0.54 (0.23–1.23)	0.05	0.05 (0.04–0.05)
In Pb Cluster	22	7.1 %	0.04	0.03 (0.02–0.15)	0.02	0.01 (0.01–0.05)	0.42	0.39 (0.24–0.74)	0.05	0.04 (0.04–0.05)
Age at Delivery										
18–24	98	31.6 %	0.03	0.03 (0.01–0.07)	0.02	0.03 (0.01–0.06)	0.40	0.40 (0.16–0.94)	0.05	0.05 (0.04–0.06)
25–34	161	51.9 %	0.02	0.02 (0.01–0.04)	0.03	0.04 (0.01–0.08)	0.35	0.29 (0.16–0.74)	0.05	0.04 (0.04–0.05)
35+	51	16.5 %	0.02	0.02 (0.01–0.04)	0.03	0.04 (0.02–0.07)	0.42	0.39 (0.17–1.07)	0.05	0.04 (0.04–0.05)
Race/Ethnicity										
Black	110	35.5 %	0.03	0.03 (0.01–0.06)	0.03	0.04 (0.01–0.07)	0.35	0.33 (0.16–0.79)	0.05	0.04 (0.04–0.05)
Hispanic	98	31.6 %	0.03	0.02 (0.01–0.09)	0.02	0.03 (0.01–0.06)	0.47	0.50 (0.22–1.07)	0.05	0.04 (0.04–0.05)
Other	12	3.9 %	0.03	0.03 (0.01–0.08)	0.05	0.05 (0.03–0.13)	0.49	0.55 (0.27–1.02)	0.06	0.05 (0.04–0.10)
White	90	29.0 %	0.02	0.02 (0.01–0.03)	0.03	0.03 (0.02–0.08)	0.32	0.21 (0.13–0.62)	0.05	0.04 (0.04–0.05)
Years of Education										
1–11 year	100	32.6 %	0.03	0.03 (0.01–0.10)	0.02	0.02 (0.01–0.05)	0.51	0.53 (0.23–1.01)	0.05	0.05 (0.04–0.05)
12 years	64	20.8 %	0.03	0.03 (0.01–0.07)	0.03	0.03 (0.01–0.06)	0.34	0.29 (0.18–0.61)	0.05	0.04 (0.04–0.05)
13–15 years	41	13.4 %	0.03	0.03 (0.02–0.06)	0.04	0.05 (0.02–0.08)	0.36	0.25 (0.16–0.85)	0.05	0.04 (0.04–0.05)
16+ yrs	102	33.2 %	0.02	0.01 (0.01–0.03)	0.04	0.04 (0.02–0.09)	0.30	0.27 (0.12–0.64)	0.05	0.04 (0.04–0.05)
Pre-pregnancy BMI (kg/m^2^)										
<18.5	12	3.9 %	0.03	0.03 (0.01–0.07)	0.04	0.04 (0.02–0.08)	0.29	0.24 (0.15–0.83)	0.05	0.04 (0.04–0.05)
18.5–24.9	118	38.4 %	0.02	0.02 (0.01–0.04)	0.03	0.04 (0.01–0.09)	0.38	0.30 (0.14–0.97)	0.05	0.04 (0.04–0.05)
25–29.9	98	31.9 %	0.03	0.03 (0.01–0.09)	0.02	0.03 (0.01–0.07)	0.40	0.43 (0.20–0.82)	0.05	0.04 (0.04–0.05)
30–34.9	45	14.7 %	0.02	0.02 (0.01–0.04)	0.03	0.04 (0.02–0.07)	0.28	0.27 (0.15–0.51)	0.05	0.04 (0.04–0.05)
35+	34	11.1 %	0.02	0.03 (0.01–0.04)	0.02	0.03 (0.01–0.05)	0.50	0.40 (0.17–1.34)	0.05	0.04 (0.04–0.05)
Whole Blood Folate (ug/L)										
low: 0–188	100	32.5 %	0.03	0.03 (0.01–0.10)	0.02	0.03 (0.01–0.06)	0.38	0.37 (0.17–0.86)	0.05	0.04 (0.04–0.06)
medium: 188–263	104	33.8 %	0.03	0.02 (0.01–0.06)	0.03	0.04 (0.01–0.08)	0.43	0.36 (0.20–0.84)	0.05	0.04 (0.04–0.05)
high: 263–358	104	33.8 %	0.02	0.02 (0.01–0.03)	0.04	0.04 (0.02–0.09)	0.33	0.25 (0.14–0.72)	0.05	0.04 (0.04–0.05)
Smoking Status										
Did not smoke during pregnancy	254	84.7 %	0.02	0.02 (0.01–0.04)	0.03	0.04 (0.01–0.08)	0.37	0.33 (0.15–0.78)	0.05	0.04 (0.04–0.05)
Smoked during pregnancy	46	15.3 %	0.05	0.04 (0.03–0.10)	0.02	0.03 (0.01–0.05)	0.38	0.30 (0.18–0.99)	0.05	0.04 (0.04–0.06)
Leisure Time Physical Activity										
No	77	25.5 %	0.03	0.02 (0.01–0.07)	0.02	0.02 (0.01–0.07)	0.39	0.37 (0.18–0.85)	0.05	0.04 (0.04–0.05)
Yes	225	74.5 %	0.02	0.02 (0.01–0.04)	0.03	0.04 (0.01–0.07)	0.37	0.31 (0.16–0.82)	0.05	0.04 (0.04–0.05)

^a^The geometric mean was calculated using values of the limit of detection (LOD) divided by the square root of 2 when < LOD

Among the 310 women for whom blood concentrations were measured, the median levels and interquartile ranges (μg/dL) for all participants were Cd 0.02 (0.01–0.04), Hg 0.03 (0.01–0.07), Pb 0.34 (0.16–0.83), and As 0.04 (0.04–0.05). A sizable proportion of women have metals concentrations more than four times higher than the median levels (Cd: 16.7 %; Hg: 11.3 %; Pb: 14.8 %; and As 2.6 %).

### Spatial distribution of elevated blood heavy metals

**Fig. 1:**
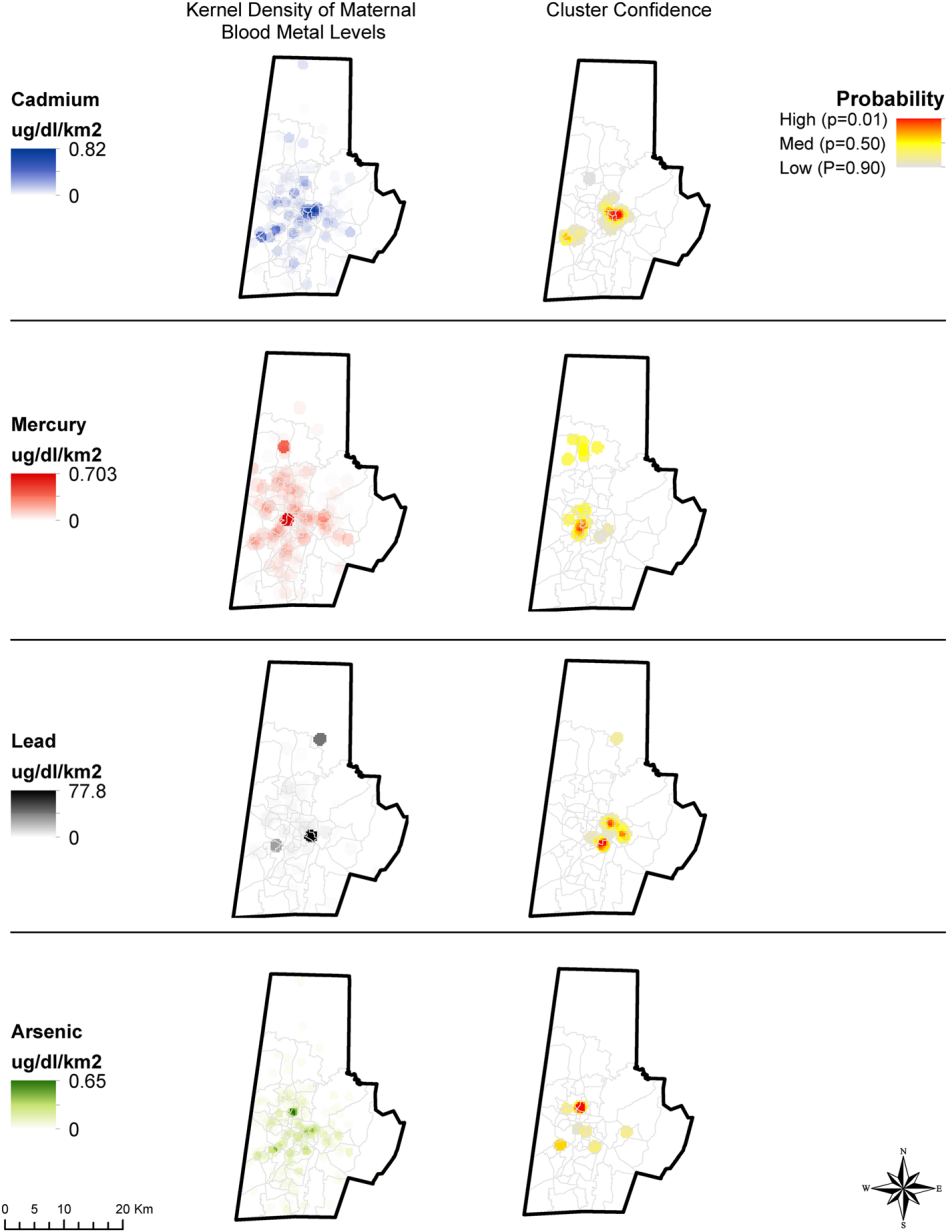
Geospatial clustering of maternal blood metal concentrations. Measured maternal blood metal concentrations (as density, ng/g/km2 cluster analysis (Gi* *p*-value) for Cd, Hg, Pb, and As. Z-scores are calculated using the Hot Spot Analysis Tool and Getis-Ord statistics (Gi* = Z-score, *p*-value) with confidence level indicated by the Gi* *p*-value. Cluster confidence is reported using a heat map representation of *p*-values, with red indicating highest confidence (*p* < 0.01) in the existence of a geographic cluster

Figure 1 shows results of the geospatial cluster analysis (Getis-Ord Gi*) of blood metals concentration for each metal based on the street address during the prenatal period. The analysis revealed geospatial clusters among the 239 Durham County residents with a high (99 %) confidence. These clusters comprised 10.5 % with elevated Cd and 9.5 % with elevated Pb levels. Median blood concentrations within the cluster were higher than overall values with Cd in the Cd cluster of 0.06 μg/dL (IQR 0.02–0.16 μg/dL) and Pb in the Pb cluster of 0.39 μg/dL (IQR 0.24–0.74 μg/dL). Clusters of As and Hg were also detected (Fig. 1), however each cluster had a limited number of women with elevated levels. An additional Cd cluster was also identified, albeit with lower confidence (Fig. 1). To evaluate the effects of population mobility during the course of pregnancy, we repeated Fig. 1 analyses in the subset of 178 Durham County women in whom addresses remained unchanged since their last menstrual period, and the results remained unaltered (not shown).

**Fig. 2:**
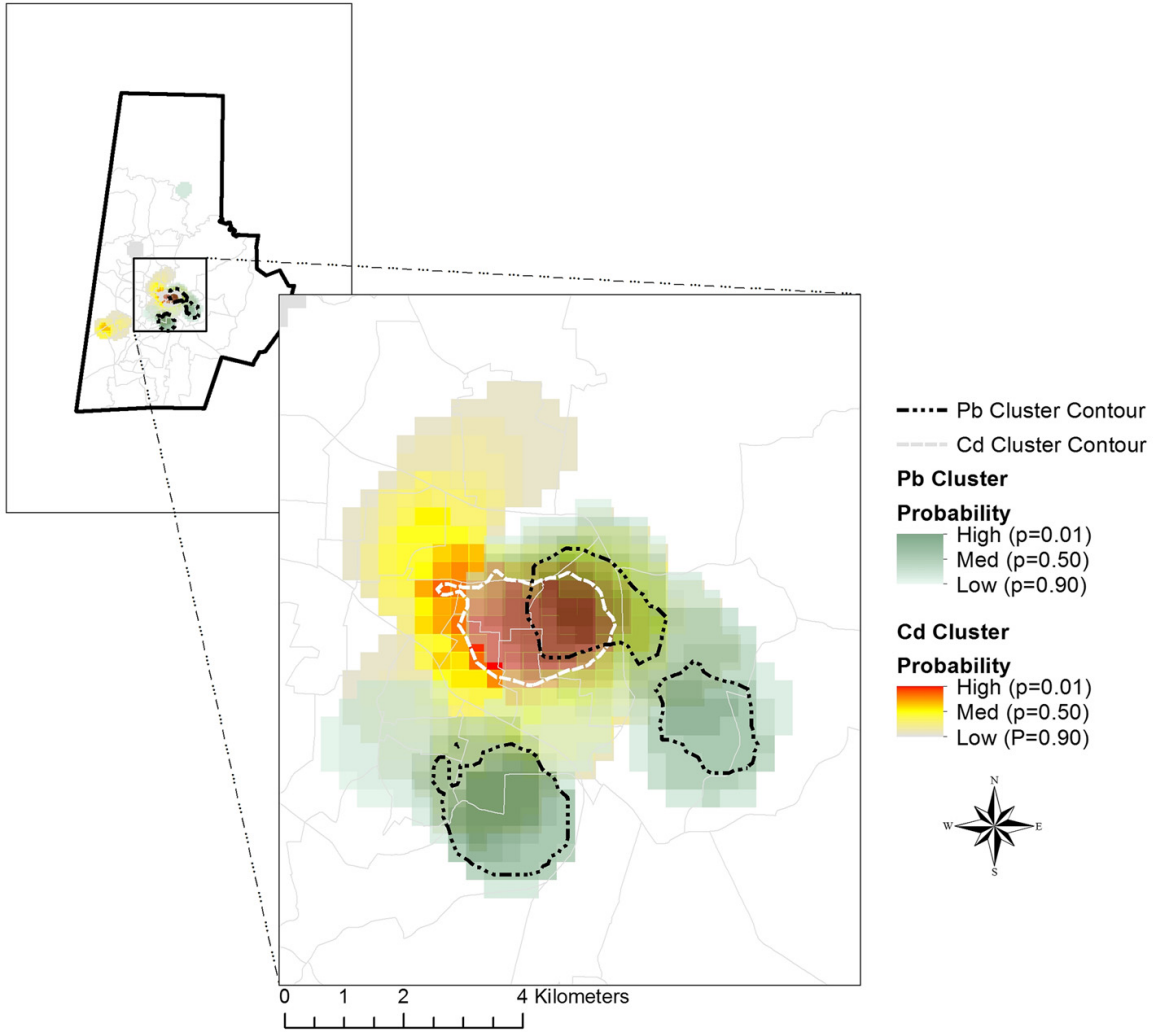
Cluster overlay for Cd and Pb. The white contour line represents the area of the highest probably of Cd clustering, while the black contour lines represent the highest probability of Pb clustering. The overlapping areas indicate high probability of both Pb and Cd clustering

Intriguingly, the Cd cluster overlapped other areas that appeared to be clusters of elevated Pb and As. Figure 2 demonstrates the overlap of the highest probability Cd cluster outlined in white with the highest probability Pb cluster outlined in black. The overlapping areas indicate high probability of both Pb and Cd clustering (*p <* 0.01). The co-occurrence of Cd, Pb and As in Figs. 1 and 2 is further corroborated by Spearman correlation coefficients between Pb and Cd exposure which are r = 0.40 (*p <* 0.001), while those between Cd and As were r = 0.21 (*p =* 0.0002), and the coefficients for As and Pb were r = 0.21 (*p* = 0.0002). Consistent with Fig. 1, in individuals, the correlation between Hg and Cd, Pb or As was inverse (r = −0.29, *p <* 0.0001, r = −0.48, *p =* <0.001, and r = −0.26, *p <* 0.001, respectively).

### Factors associated with heavy metal blood levels

Table 2 shows regression coefficients for the relationship between the metal values of Cd, Pb, Hg, and As (categorized into tertiles) and sociodemographic and lifestyle correlates: education, obesity, folate levels, smoking, and physical activity. Consistent with previous studies, we found that elevated Cd levels were associated with cigarette smoking during pregnancy (OR = 22.43, *p <* 0.0001 at the highest tertile of Cd) and fewer years of education (OR = 0.83, *p =* 0.0001 at the highest tertile of Cd). However, a higher level of education was associated with higher Hg levels (OR = 1.16, *p* = 0.0008 at the highest tertile of Hg) while no physical activity during pregnancy was associated with decreased Hg levels (OR = 0.57 *p* = 0.0787 at the highest tertile of Hg). Higher education levels were also associated with lower Pb (OR = 0.90, *p* = 0.0242 at the highest tertile of Pb) and As (OR = 0.88, *p* = 0.0048 at the highest tertile of As).

**Table 2:** Associations between metal levels and sociodemographic and lifestyle factors

Factor	Metal Exposure level	Cadmium (Cd)	Mercury (Hg)	Lead (pb)	Arsenic (As)
	Reference = low	OR (95 % CI), *p*-value	OR (95 % CI), *p*-value	OR (95 % CI), *p*-value	OR (95 % CI), *p*-value
		*N* = 300	*N* = 300	*N* = 297	*N* = 300
Smoking Status (Reference = did not smoke during pregnancy)	Medium	5.94 (1.27–27.76), 0.0235	0.98 (0.45–2.15), 0.9578	0.91 (0.39–2.14), 0.8333	1.53 (0.66–3.56), 0.3266
	High	22.43 (5.02–100.19), <0.0001	0.54 (0.23–1.3), 0.1675	1.81 (0.79–4.16), 0.1612	1.58 (0.71–3.53), 0.2615
		*N* = 308	*N* = 308	*N* = 305	*N* = 308
Whole Blood Folate (ug/L)	Medium	1.00 (1.00–1.00), 0.6557	1.01 (1.00–1.01), 0.0062	1.00 (0.99–1.00), 0.1860	1.00 (1.00–1.00), 0.8695
	High	1.00 (0.99–1.00), 0.0091	1.01 (1.00–1.01), 0.0018	1.00 (0.99–1.00), 0.3416	1.00 (1.00–1.00), 0.9863
		*N* = 302	*N* = 302	*N* = 300	*N* = 302
Leisure-time Physical Activity (Reference = yes)	Medium	0.62 (0.32–1.19), 0.1504	0.44 (0.23–0.86), 0.0158	1.00 (0.51–1.95), 0.9968	0.57 (0.29–1.11), 0.0994
	High	0.73 (0.38–1.39), 0.3355	0.57 (0.30–1.07), 0.0787	1.18 (0.6–2.31), 0.6306	0.81 (0.43–1.53), 0.5177
		*N* = 307	*N* = 307	*N* = 304	*N* = 307
Years of Education	Medium	0.93 (0.86–1.01), 0.0993	1.05 (0.96–1.15), 0.2633	0.93 (0.85–1.01), 0.0964	0.91 (0.83–1), 0.0380
	High	0.83 (0.76–0.91), 0.0001	1.16 (1.07–1.27), 0.0008	0.90 (0.82–0.99), 0.0242	0.88 (0.8–0.96), 0.0048
		*N* = 307	*N* = 307	*N* = 304	*N* = 307
Pre-pregnancy BMI (kg/m^2^)	Medium	1.02 (0.98–1.06), 0.4387	1.01 (0.97–1.05), 0.5923	1.01 (0.96–1.05), 0.7774	1.02 (0.98–1.06), 0.3721
	High	1.00 (0.96–1.05), 0.9692	0.98 (0.93–1.02), 0.2855	1.01 (0.97–1.06), 0.5335	0.99 (0.95–1.03), 0.5995

Covariates entered in separate models. All models adjusted for maternal race/ethnicity and age

Table 3 shows the ORs for the association between socio-demographic factors and Cd or Pb cluster membership, adjusted for maternal education, pre-pregnancy BMI, folate concentrations (μg/L), and smoking status. All cluster members were Hispanic or non-Hispanic Blacks. The only factors associated with cluster membership were lower educational level (OR = 0.84, 95 % confidence intervals = 0.75–0.95, *p* = 0.0043 for Cd and OR = 0.87, 95 % confidence intervals = 0.78–0.98, *p* = 0.0204 for Pb) and higher pre-pregnancy BMI (OR = 1.06, 95 % confidence intervals = 0.99–1.13, *p* = 0.0983 for Cd and OR = 1.07, 95 % confidence intervals = 1.00–1.14, *p* = 0.0401 for Pb).

**Table 3:** Predictors of Cadmium (Cd) and Lead (Pb) cluster membership

	Cadmium (*n* = 227) OR (95 % CI), *p*-value	Lead (*n* = 227) OR (95 % CI), *p*-value
Education	0.84 (0.75–0.95), 0.0043	0.87 (0.78–0.98), 0.0204
Whole Blood Folate (ug/L)	1.00 (0.99–1.00), 0.5018	1.00 (1.00–1.01), 0.6530
Pre-pregnancy BMI (kg/m^2^)	1.06 (0.99–1.13), 0.0983	1.07 (1.00–1.14), 0.0401
Smoking Status (Reference = did not smoke during pregnancy)	0.99 (0.27–3.67), 0.9877	0.60 (0.13–2.77), 0.5080

No cluster members were non-Hispanic White therefore we did not adjust for race

### Cd and Pb in soils and water

To identify possible sources of Pb and Cd, we evaluated the relationship of Pb and Cd in city water, soil and blood concentrations among 24 households of women with Cd levels ≥ 0.1 ng/g (0.01035 μg/dL) and 17 with Cd levels < 0.1 ng/g. There were no detectable levels of Pb or Cd in city water, whereas levels in soil ranged from 0.0–1.6 mg/kg for Cd (0.1 median, 0.1–0.2 interquartile range) and 2–212.5 mg/kg for Pb (19.9 median, 8.8–38.7 interquartile range) (Table 4). Consistent with the significant correlation between blood Pb and Cd, we also found a significant Spearman correlation between soil Pb and Cd (r = 0.55, *p =* 0.0002) suggesting co-contamination. Soil Pb levels were also significantly correlated with blood Pb levels (r = 0.31, *p =* 0.0534). The correlation between blood Cd and soil Cd was not significant (r = 0.009, *p* = 0.9556).

**Table 4:** Levels of Cd and Pb in Soil (mg/kg)

	Metal	N	Mean	S.D.	Minimum	25th Percentile	Median	75th Percentile	Maximum
Overall	Cd	41	0.19	0.27	0.00	0.06	0.11	0.19	1.58
	Pb	41	39.64	53.11	2.03	8.78	19.93	38.72	212.51
Cadmium blood levels <1	Cd	17	0.17	0.15	0.00	0.06	0.14	0.27	0.49
	Pb	17	40.32	53.09	2.03	6.89	29.26	39.74	212.51
Cadmium blood levels ≥1	Cd	24	0.21	0.33	0.00	0.06	0.11	0.19	1.58
	Pb	24	39.16	54.25	4.38	9.63	16.46	32.66	181.91
Reference [1, 59]	Cd	-	0.06	-	0.01	-	-	-	0.70
	Pb	-	10	-	2	-	-	-	200

## Discussion

We determined if concentrations of four metals in the peripheral blood of pregnant women living in urban Durham County, NC clustered spatially. We also identified factors associated with elevated metal concentrations and clustering. We further explored the extent to which water or soil was a possible source of the metal contamination.

Our key findings were that Cd, Pb and As co-occurred in blood of pregnant women whereas women with elevated Hg concentrations were more likely to have lower Cd, Pb and As. Furthermore, women in an inner city neighborhood were clustered with higher concentrations of Cd and Pb in comparison to the rest of the study area. Cluster membership was associated with a lower educational level and higher pre-pregnancy BMI; all cluster members were Hispanic or non-Hispanic Black. Cadmium blood concentrations within the Cd cluster were notably higher than levels reported among third trimester pregnant women from 6 counties in North Carolina, among pregnant women at delivery in Durham County in a previous study, and the US population age 20 years and older based National Health and Nutrition Examination Study (NHANES) [34, 36, 37]. Reference and reportable values for blood metals levels are given in Table 5. Similar or lower blood concentrations of Cd in pregnant women have been associated with lower birth weight [55], a consistent risk factor for common chronic diseases and conditions [56]. These data support that spatial analysis of blood concentrations of heavy metals can aid in focusing limited screening resources to smaller sub-populations with highest risk of exposure.

**Table 5:** Reference and reportable values for blood metals levels (μg/dL)

Population	Timeframe	Measure	Cd	Hg	Pb	As
US age 20+ yrs [34]	2011–2012	Geometric Mean (95 % CI)	0.0337 (0.0323–0.0353)	0.0863 (0.0753–0.0990)	1.09 (1.03–1.16)	N/A
New York City age 20+ yrs [35]	2004	Geometric Mean (95 % CI)	0.077 (0.075–0.080)	0.273 (0.258–0.289)	1.79 (1.73–1.86)	N/A
Pregnant women (3^rd^ trimester) in NC [36]	2009–2011	Geometric Mean (range)	0.0181 (<0.011–0.279)	0.0453 (<0.023–1.178)	0.890 (0.19–7.72)	0.0445 (<0.023–0.858)
Pregnant women (delivery) in Durham County [37]	2005–2010	Geometric Mean (range)	0.033 (0–0.402)	N/A	N/A	N/A
New York State [38]	n/a	Reportable Value	1	0.5	All	N/A
Michigan State [39]	n/a	Reportable Value	0.5	1.5	5	7
OSHA [40]	n/a	BEI^a^	0.5	1.5	30	N/A
CDC [41]	n/a	Reportable Value for Children and Pregnant Women	N/A	N/A	5	N/A

^a^The Biological Exposure Incides (BEI) are published by the American Conference of Governmental Industrial Hygienists (ACGIH). It is intended to serve as a general guideline for industrial hygienists evaluating biomonitoring results [67]

Whereas elevated Pb levels have been repeatedly described in pregnant women, few studies have examined its co-occurrence with other heavy metals, whose interaction may contribute to future chronic disease risk. The overall geometric mean for blood Arsenic was comparable to the geometric mean found previously among pregnant women in NC [36]. The geometric means for Hg and Pb were lower than previously reported values, however Pb values for 23 % and Hg values for 41 % of women were higher than the geometric mean previously reported for pregnant women in NC [36].

While the small sample size limits inference, our data support that long-term low dose exposure to multiple heavy metals can be monitored routinely in blood commonly collected and discarded, especially during pregnancy and sub-populations at high risk for exposure to heavy metals can be identified using geospatial analysis. These data also reinforce the hypothesis that heavy metal exposure in pregnant women can be clustered in an inner city neighborhood [57, 58]. This interdependence of lower socioeconomic status and elevated metal levels often complicates interpretation of etiologic studies aimed at relating such exposure to chronic diseases that are also common in these populations.

The mean level of heavy metals in the soil was above the average 0.06 for Cd and 10 mg/kg for Pb found elsewhere [1, 59]. All measured values of Pb and Cd in our soil samples were below the EPA recommended level for cleanup of 400 mg/kg for Pb and 70 mg/kg for Cd [1, 59]. Much like recommendations for blood level heavy metals, the maximum level of metals in the soil also varies with the New York State Brownfield Cleanup program recommending a significantly lower maximum of 0.86 mg/kg for Cd in Soil of residential areas [60].

Our data also suggest that Pb and Cd co-occur in the environment and in individuals. We found co-occurrence with Pb and Cd in soil but not in water; blood levels of Pb but not Cd were significantly positively correlated with soil concentrations, suggesting that the source of contamination is likely to be local soil. Soil contamination with these toxins has also been previously reported in urban and rural landscapes [3–5]. While geochemical fingerprinting and measurement of bioaccessible fractions of heavy metals was beyond the scope of these analyses, these data support that the likely routes are ingestion or inhalation of aerosolized house dust. If confirmed in larger studies, these data can support the development of intervention studies to generate data for policy decisions.

Cd and Pb cluster membership was associated with lower socioeconomic status (SES)—estimated by lower educational status and higher pre-pregnancy BMI. Education and race/ethnicity may either influence residential preference or the wherewithal to reside at geographic locations outside the cluster, or may be related to dietary heavy metals consumption. For instance, the higher levels of Hg (not measured in the soil or water) in more educated women may be an unintended consequence of increasing fish consumption because fish oil may benefit brain development during gestation [61]. Among Spaniards [62] Pb and Cd concentrations were 15 and 22 % higher in newborns from mothers who smoked during pregnancy, while Hg concentrations were 25 % higher in newborns from mothers with greater fish intake. Thus, SES may certainly play a direct role in influencing dietary metals exposure, and an indirect role by influencing residential proximity to contaminated soils, or both.

Consistent with this view, there were no differences between cluster members and non-members in cigarette smoking. Folate levels could be capturing different aspects of SES. For instance, disadvantaged groups are less likely to have a diet replete with folate [63] and perhaps other B vitamins that co-occur in the diet. Indeed folate and other B vitamins are critical to the generation of S-adenosyl-methionine (SAM)-the universal methyl group donor—and SAM is key in the biotransformation of As. Changes in SAM availability due to As exposure can alter epigenetic response [64]. Dietary depletion of one carbon cycle nutrients reduces As excretion and methylation [42, 65], and repletion of these nutrients lowers blood As concentrations in human adults [44]. Discerning the underlying risk factors for heavy metal contamination will require clarifying these relationships.

While the traditional matrix for estimating the burden of metals such as Cd in humans is urine or feces, because long term storage is either in the kidney cortex and bile – where long term exposure is often estimated; measurement in blood has the ability to provide estimates of both concurrent exposure and exposure mobilized from long term storage including tissue and bone [47–50, 66]. As some heavy metals including Pb and Cd attach to erythrocytes, and the lifespan of erythrocytes is ~120 days, specimens that were collected at ~12 weeks gestation provide data on heavy metal exposure during a rarely evaluated developmental window, the periconceptional during which many metabolic set points are established. A limitation of our study is the modest sample size, which precludes analysis of metal mixtures such as As and Hg, which also may co-occur in soil, water and blood. It is likely that metal mixtures, even at low levels, could interact to influence future chronic disease susceptibility. In addition, while Pb and Cd could be measured in soils, we could not cost-effectively multiplex As and Hg using the available instrumentation. Despite these limitations, these data support that commonly collected blood specimens can be a useful matrix for monitoring the spatial distribution of exposure to heavy metals in urban landscapes where traditional risk assessment strategies are cost-prohibitive.

## Conclusions

Our data are consistent with the emerging view that Cd, Pb, Hg, and As are common exposures. Chronic co-exposure, even at low doses, increases the risk of low birth weight, a consistent risk factor for cardiometabolic diseases, neurological diseases and some cancers in later life. Spatial analysis can be a useful tool to identify locations where patient populations may be at risk of exposure, making early intervention possible for reducing the risk of further chronic diseases. With no effective chelating agents for low dose chronic exposure, research on early identification of exposed populations is greatly needed, as environmental remediation may be slow and costly. Larger studies are required to confirm these findings.
